# Alignment of Tractograms As Graph Matching

**DOI:** 10.3389/fnins.2016.00554

**Published:** 2016-12-05

**Authors:** Emanuele Olivetti, Nusrat Sharmin, Paolo Avesani

**Affiliations:** ^1^NeuroInformatics Laboratory, Bruno Kessler FoundationTrento, Italy; ^2^Center for Mind and Brain Sciences, University of TrentoTrento, Italy

**Keywords:** diffusion MRI, tractography, alignment, combinatorial optimization, graph matching

## Abstract

The white matter pathways of the brain can be reconstructed as 3D polylines, called streamlines, through the analysis of diffusion magnetic resonance imaging (dMRI) data. The whole set of streamlines is called tractogram and represents the structural connectome of the brain. In multiple applications, like group-analysis, segmentation, or atlasing, tractograms of different subjects need to be aligned. Typically, this is done with registration methods, that transform the tractograms in order to increase their similarity. In contrast with transformation-based registration methods, in this work we propose the concept of tractogram correspondence, whose aim is to find which streamline of one tractogram corresponds to which streamline in another tractogram, i.e., a map from one tractogram to another. As a further contribution, we propose to use the relational information of each streamline, i.e., its distances from the other streamlines in its own tractogram, as the building block to define the optimal correspondence. We provide an operational procedure to find the optimal correspondence through a combinatorial optimization problem and we discuss its similarity to the graph matching problem. In this work, we propose to represent tractograms as graphs and we adopt a recent inexact sub-graph matching algorithm to approximate the solution of the tractogram correspondence problem. On tractograms generated from the Human Connectome Project dataset, we report experimental evidence that tractogram correspondence, implemented as graph matching, provides much better alignment than affine registration and comparable if not better results than non-linear registration of volumes.

## 1. Introduction

Diffusion magnetic resonance imaging (dMRI) data (Basser et al., 1994), provide quantitative information about the white matter of the brain, in terms of local main direction(s) of the neuronal axons. Such information allows to approximate the main paths of large sets of axons with polylines, called streamlines. The whole set of streamlines is called tractogram and represents the structural connectome of the brain (Sporns et al., 2005).

In multiple applications, like group-analysis, segmentation, or atlasing, tractograms of different subjects need to be aligned (O'Donnell et al., 2012), i.e., it is necessary to find corresponding anatomical structures across tractograms. Typically, two tractograms can be aligned by first estimating a transformation between the corresponding volumetric images, like T1, fractional anisotropy (FA, see Basser et al., 1994), or orientation distribution functions (ODFs, see Raffelt et al., 2011; Christiaens et al., 2012, and then by applying such transformation to the streamlines of the tractograms^1^. In the literature, both linear and non-linear methods have been proposed to register volumetric data, see Christiaens et al. (2014). In case of linear methods, an affine transformation is estimated, e.g., with FSL/FLIRT, see Jenkinson et al. (2002), and then applied to the coordinates of the streamlines. In case of non-linear volumetric transformations, the tractogram is usually re-generated after transforming the dMRI data accordingly. The literature on non-linear transformations directly on streamlines is focused on fiber bundle alignment. In Ziyan et al. (2007), corresponding bundles are aligned across subjects by warping their spatial probability maps computed from the coordinates of the streamlines. In Ziyan et al. (2009), consistency clustering is used to label streamlines in an iterative process with polyaffine transformations. In Wassermann et al. (2011) a diffeomorphic registration algorithm is used on the Gaussian process representation of streamline density maps. In Durrleman et al. (2011), the use of the framework of *currents* is proposed to warp the streamlines of bundles, for registration, atlasing, and variability analysis. This literature addresses non-linear bundle alignment but not whole tractogram alignment, also for the high computational cost of the algorithms. Another approach to non-linear registration of streamlines is to use deformation fields, computed from non-linear volume registration, directly on streamlines. This approach is mentioned in Christiaens et al. (2012), but without a quantitative evaluation of its effect.

Recently, new linear methods have been proposed to directly register whole tractograms, see O'Donnell et al. (2012) and Garyfallidis et al. (2015), without the intermediate indirect step of registering volumetric images. These methods find an affine transformation that minimizes a given loss function computed only from streamlines. In O'Donnell et al. (2012), the loss function is based on the entropy of a random subset of the coordinates of the streamlines. In Garyfallidis et al. (2015), the loss function is based on a streamline-streamline distance of a subset of all streamlines.

**Figure 1:**
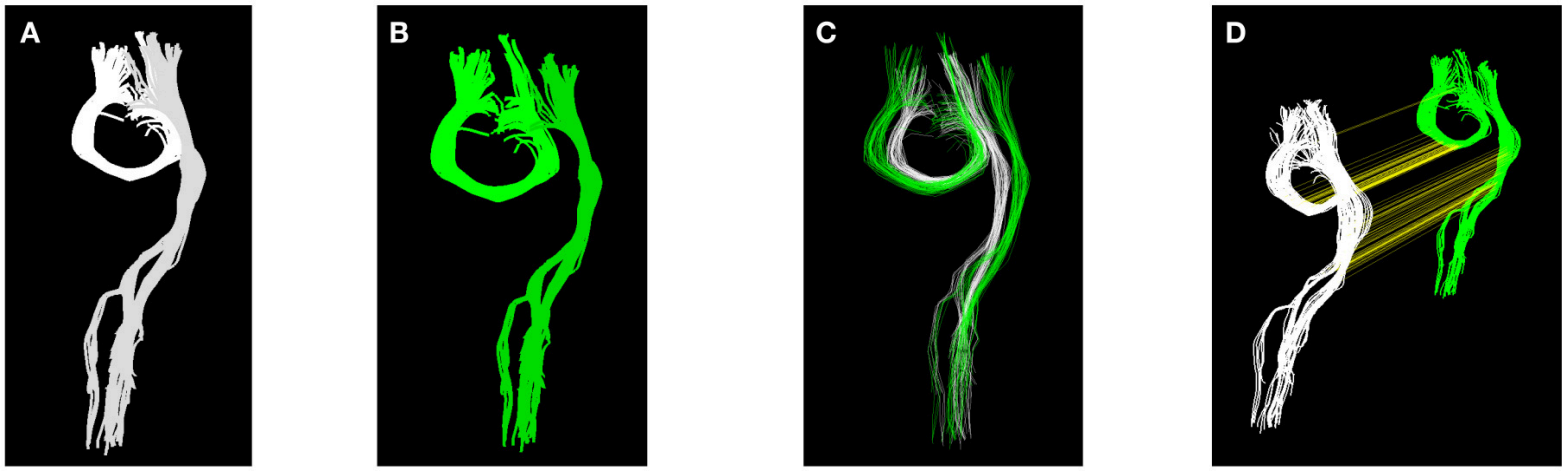
**Panels (A,B)** show two equivalent sets of streamlines, with local differences. Panel **(C)** shows the effect and limits of linear registration of panels **(A,B)**. Panel **(D)** shows the result of the proposed tractogram correspondence, where streamlines of panel **(A)** are connected with a yellow straight line to the corresponding ones of **(B)**.

It is common experience that a single affine transformation can reconcile only part of the differences between the tractograms of two subjects. Many differences remain at the local level, e.g., systematic displacement of entire tracts, thinner/thicker, or longer/shorter tracts. We show a simple toy example of these issues in Figure 1, where two tracts (see Figure 1A), one U-shaped and the other one elongated, and the corresponding two tracts after some local changes (see Figure 1B), are presented. This second set of streamlines, (B), is artificially constructed from the first set (A) by adding a small displacement between the two tracts and a moderate magnification of the U-shaped one. By construction it is not possible to find a global affine transformation that reconciles the difference between A and B, because the difference is *local* and not global. In Figure 1C we show the overlap between (A) and (B) with the best affine transform obtained with the streamline linear registration (SLR, see Garyfallidis et al., 2015) algorithm. It is clearly visible that the overlap between the green (A) and white (B) sets is poor.

As in O'Donnell et al. (2012) and in Garyfallidis et al. (2015), in this work we are eminently interested in methods for tractogram alignment that directly operates on streamlines, without resorting to volume-based registration. This is due to two main reasons: first, in many practical cases, tractogram alignment is based on registration of images that either do not contain diffusion MRI information at all, i.e., T1 images, or contain just a portion of it, e.g., FA images, which is clearly suboptimal. In case full ODFs are considered (see for example Raffelt et al., 2011), the effects of re-orientation may create problems when re-generating the tractogram, see Christiaens et al. (2012). The second reason is that the cost of re-generating tractograms, after registering volumes, may be high, in terms of computation time and required knowledge to operate the full pipelines of, preprocessing, reconstruction, tracking, and filtering, see Pestilli et al. (2014).

In this work, we propose to avoid transformations and to directly find the correspondence between streamlines of two different tractograms, i.e., to find which streamline of one tractogram corresponds to which streamline in the other tractogram. This idea is inspired by our recent work on bundle segmentation (Sharmin et al., 2016). The concept of corresponding streamlines has been used before, for example in the context of streamline clustering. There, the aim is to find corresponding clusters across subjects or to label them from an atlas. The cluster correspondence is achieved by finding the correspondence between the representative streamlines, one per cluster. See for example Maddah et al. (2005), O'Donnell and Westin (2007), Guevara et al. (2012), and Yoo et al. (2015). In this work, we take one step further and target streamline correspondence for all streamlines in the tractogram. This can be thought as the limit case of cluster correspondence, when the size of the cluster decreases till one single streamline. It is true that, given two tractograms, we cannot assume that *every* streamline in one tractogram has a corresponding streamline in the other tractogram. This occurs because, for example, some streamlines could be artifactual and have no anatomical counterpart. Nevertheless, in Section 3, show that, in practice, such cases do not disrupt the quality of the resulting alignment and that, for this reason, the number of problematic streamlines is quite small.

The alignment based on streamline correspondence, in a sense, acts as a non-linear alignment of the first set of streamlines onto the second one. It can be seen as a transformation, where each streamline of one tractogram is moved and deformed to become of the exact shape and position of the corresponding streamline in the other tractogram. From this point of view, the proposed method can be thought as an extremely local registration method. In Figure 1D, we show the result of computing such correspondence. There, the two sets of streamlines are displayed with arbitrary displacement and yellow straight lines, found by the proposed algorithm, correctly connect the midpoints of the corresponding streamlines across the two sets. After alignment, the resulting aligned set of white streamlines will be identical to the set of green streamlines.

Alignment based on streamline correspondence provides immediate means to address practical tasks such as bundle segmentation and streamline labeling. Once the label of a streamline is known in one subject, then such label can be directly assigned to the corresponding streamline in the tractogram of the other subject. In such applications, the interest is in transferring anatomical information to a specific target subject, for example in the context of pre-surgical planning. Notice that this aim is different from, for example, that of constructing atlases, see Durrleman et al. (2011). In that scenario the interest is to find a common model from multiple subjects and, for this reason, none of them can be considered as ground truth.

The algorithmic problem of finding the correspondence between streamlines of different tractograms can be framed as a combinatorial optimization problem. Given a loss function that measures how good such correspondence is, the task is to explore the combinatorial space of all possible correspondences in order to find the one that minimizes such loss. To define the loss function, in this work, we propose to encode each tractogram as a large fully-connected weighted graph, where each node/vertex corresponds to a streamline and the weights of the graph's edges are the distances between streamlines within the same set. Given two tractograms, i.e., two graphs, the original problem of finding the correspondence between two sets of streamlines, can be recast as the problem of finding corresponding nodes/vertices between the two graphs, a famous combinatorial optimization problem called *graph matching*, see Conte et al. (2004). In this work, we provide an operational procedure to compute tractogram alignment based on a recent approximate graph matching algorithm, i.e., the doubly stochastic fixed-point (DSPFP) algorithm.

In the following sections of this paper, we formally present the tractogram correspondence problem and explore its similarity to the graph matching problem (see Section 2), from whose literature we draw the actual algorithm adopted in our experiments. In Section 3, on dMRI data from the Human Connectome Project (HCP) dataset, see Sotiropoulos et al. (2013), we show the efficacy of tractogram correspondence, where it achieves much better alignment across subjects than standard affine-based global registration and comparable, if not better, results to non-linear voxel-based methods. We conclude this work by discussing the merits and limitations of the proposed method and by mentioning future work.

## 2. Methods

In this section, we first introduce the basic terminology and notation. Then we describe the details of tractogram correspondence in general and its implementation based on relational information. We proceed to describe the strong similarity with the problem of graph matching in order to adopt an efficient approximate solution from its literature, i.e., the DSPFP algorithm.

Let *s* = [**x**_1_, …, **x**_*n*_*s*__], ${\text{x}}_{i}\in {\mathbb{R}}^{3}$, be a *streamline* (or track, or fiber), i.e., a polyline made of a sequence of *n*_*s*_ points in 3D space. Let *T* = {*s*_1_, …, *s*_*N*_} be a *tractogram* (or track set) of the whole brain and *t* ⊂ *T* a *tract* (or bundle). Let *d*:*T* × *T* ↦ ℝ^+^ be a distance function between streamlines. Several anatomically meaningful distances have been proposed in the literature, based on the idea that streamlines with similar path and shape belong to the same anatomical structure, see Gerig et al. (2004), Corouge et al. (2004), Zhang et al. (2008), and Jiao et al. (2010). In this work we use the commonly adopted mean average minimum (MAM) distance, a modified Hausdorff distance sometimes called also mean closest point distance (see Corouge et al., 2004):

(1) $$\begin{array}{l} d_{MAM}\left(s,s^{\prime }\right)=\frac{1}{2}\left(D\left(s,s^{\prime }\right)+D\left(s^{\prime },s\right)\right) \end{array}$$

where $D\left(s,s^{\prime }\right)=\frac{1}{n_{s}}\overset{n_{s}}{\underset{i=1}{\sum }}d\left({\text{x}}_{i},s^{\prime }\right)$, and $d\left(\text{x},s^{\prime }\right)={\text{min}}_{j=1,\ldots ,n_{s^{\prime }}}||\text{x}-{\text{x}}_{j}^{\prime }|{|}_{2}$. Other distances can be used without changes to the procedure we propose here.

Let *G* = (*V, E, f*) be a fully-connected undirected edge-weighted graph, made of a finite set of vertices *V*, the corresponding set of edges *E*, and a weighting function *f*:*E* ↦ ℝ. We represent the tractogram *T* with the graph *G*: each vertex of *G* correspond^2^ to one streamline of *T* and the weight of each edge is the distance between the related streamlines, i.e., *G* = (*id*(*T*), *E, d*), where *id*(*s*_*i*_) = *i*, *id*(*T*) = {*id*(*s*_1_), …, *id*(*s*_*N*_)}, *E* ⊆ *id*(*T*) × *id*(*T*) and *f*((*i, j*)) = *d*(*s*_*i*_, *s*_*j*_). Two different tractograms *T*_*A*_ and *T*_*B*_ are then represented by two different graphs, *G*_*A*_ and *G*_*B*_. We denote as $A\in {\mathbb{R}}^{N_{A}\times N_{A}}$ and $B\in {\mathbb{R}}^{N_{B}\times N_{B}}$ the adjacency matrices of the weighted graphs *G*_*A*_ and *G*_*B*_, respectively. Given a matrix *A*, we call *a*_*ij*_ the corresponding element at row *i* and column *j*.

### 2.1. Tractogram alignment as tractograms correspondence

We cast the problem of aligning two tractograms, *T*_*A*_ and *T*_*B*_, as the problem of finding the corresponding streamlines between them. Once such correspondence has been established, *T*_*A*_ will be aligned to *T*_*B*_ by transforming each streamline $s^{A}\in T_{A}$ into the corresponding streamline $s^{B}\in T_{B}$, as if to perfectly match it. Notice that such transformation is not explicit, i.e., no deformation field is computed. Knowing *s*^*B*^, i.e., knowing its coordinates, is enough obtain the aligned *s*^*A*^.

In order to solve the correspondence problem, we define the binary matrix $Q={\left[q_{ik}\right]}_{ik}\in {\left\{0,1\right\}}^{N_{B}\times N_{A}}$, that we call *correspondence matrix*, such that *q*_*ik*_ is 1 when streamline *i* of *T*_*A*_ corresponds to streamline *k* of *T*_*B*_ and 0 otherwise. We require that $\underset{k}{\sum }q_{ik}=1$, i.e., each streamline in *T*_*A*_ must have a corresponding one in *T*_*B*_, while the contrary is not necessarily true. This last case, for example, occurs when *N*_*A*_ ≠ *N*_*B*_, i.e., when the one-to-one correspondence cannot be made. In particular, when *N*_*A*_ > *N*_*B*_ multiple streamlines of *T*_*A*_ may correspond to the same streamline of *T*_*B*_.

In the meanwhile, we notice that the discrete space on which the loss function needs to be minimized is extremely large, i.e., there are $N_{B}^{N_{A}}$ different possible correspondences. Given the typical size of tractograms, in the order of 10^5^ streamlines, it is apparent that the combinatorial optimization problem is extremely hard to solve and that approximations should be introduced (see Section 2.4).

### 2.2. Correspondence based on relational information

Finding the best correspondence between two tractograms requires two ingredients: the definition of a loss function, i.e., a function that scores the correspondence, and the exploration of the combinatorial space in order to actually find the best correspondence. Notice that the definition of a loss function for tractogram correspondence should not be based on the direct distance between streamlines across different tractograms, because such distance becomes anatomical meaningful only *after* the alignment is done. For this reason, as the building block of the loss function, we propose that two streamlines of different tractograms should correspond when their respective set of distances, with other streamlines in their own tractograms, are similar. This idea is motivated by our results in Olivetti et al. (2012), where it was shown that representing a streamline as the set of distances from the other streamlines in its own tractogram is indeed an accurate Euclidean embedding, i.e., the geometrical information of the streamlines is preserved.

Formally, if *Q* is a correspondence matrix of *T*_*A*_ to *T*_*B*_, then the matrix $QAQ^{⊤}\in {\mathbb{R}}^{N_{B}\times N_{B}}$ represents *A* after applying such correspondence. To better understand this step, consider that, in the special case where *N*_*A*_ = *N*_*B*_ and *Q* is a one-to-one correspondence, then *Q* is a permutation matrix and *QAQ*^⊤^ is a matrix where rows and columns are obtained just by permuting those of *A* according to *Q*. As a consequence, a meaningful definition of distance, i.e., loss *L*, between *T*_*A*_ and *T*_*B*_ is the matrix distance (Frobenius norm, || · ||_*F*_) between the mapped *A* and *B*: $L=||B-QAQ^{⊤}|{|}_{F}^{2}$. Then, the problem of finding the best possible correspondence Q^*^ between *T*_*A*_ and *T*_*B*_ becomes the combinatorial optimization problem

(2) $$\begin{array}{l} Q^{*}=\underset{Q\in Q}{\text{argmin}}||B-QAQ^{⊤}|{|}_{F}^{2} \end{array}$$

where Q is the space of all possible correspondences. As noted before, Q is extremely large and, clearly, this problem is extremely hard to solve. In the following, we describe its similarity with another combinatorial optimization problem, i.e., graph matching, in order to draw approximate solutions from its literature.

### 2.3. Graph matching

A combinatorial optimization problem, that is strongly related to the tractogram correspondence problem, is *graph matching* (GM) (see Conte et al., 2004; Zaslavskiy et al., 2009). The GM problem aims at finding the corresponding vertices in two graphs, *G*_*A*_ and *G*_*B*_, of equal size, with adjacency matrices *A* and *B*. Differently from the correspondence of streamlines described in Section 2.2, the correspondence of GM is a one-to-one (bijection). Formally, the GM problem is formulated as

(3) $$\begin{array}{l} P^{*}=\underset{P\in P}{\text{argmin}}||A-PBP^{⊤}|{|}_{F}^{2} \end{array}$$

which is very similar^3^ to the correspondence problem of Equation (2). Specifically, when the correspondence between streamlines is a permutation of indices, then the matrix *Q* is a permutation matrix and the correspondence problem of Equation (2) becomes identical to the graph matching problem of Equation (3): $||A-PBP^{⊤}|{|}_{F}^{2}=||B-QAQ^{⊤}|{|}_{F}^{2}$ when *P* = *Q*, so Q^*^ = P^*^.

When the two graphs have different sizes, the GM problem is called *sub-graph matching* (sub-GM) problem and the goal is to find the corresponding vertices from the small graph to the large graph. The GM and sub-GM problems are known to be NP-hard (Conte et al., 2004), in general, and only approximate solutions can be obtained in most of the practical cases, i.e., when the number of vertices is more than twenty. Notice that a matching *P* can also be considered a correspondence, i.e., P∈P⊆Q. For this reason, an approximate solution of GM is also an approximate solution of the correspondence problem.

### 2.4. Graph matching: approximate solutions

Finding good approximate solutions to the GM and sub-GM problems is a difficult task with extensive literature. The main solutions available are divided in two groups (Zaslavskiy et al., 2009): the first group operates on spectral representations of the adjacency matrices (see for example Umeyama, 1988). The second group is based on relaxation methods (see for example Zaslavskiy et al., 2009), i.e., on solving the optimization problem on a continuous superset of P, where more efficient continuous optimization techniques can be used, and then by projecting the result back to P. To the best of our knowledge, the computational time and space complexity of the most efficient algorithms^4^ for sub-GM are, respectively, $O\left(n^{3}\right)/\text{iteration}$ and $O\left(n^{2}\right)$, reached only by the following algorithms (see Zaslavskiy et al., 2009; Lu et al., 2016): projected gradient, graduated assignment, PATH, and DSPFP. With such computational complexity, approximate solutions can be found for graphs with several thousands of vertices. To scale-up to larger graphs, it is necessary to derive methods with lower complexity per iteration or to introduce assumptions on the structure of the data.

According to Lu et al. (2016) and to our tests, the DSPFP algorithm outperforms others algorithms in terms of actual time and memory requirements, moreover with higher quality of the approximated solution. For this reason we adopted that algorithm for the experiments of Section 3. In the following we briefly review DSPFP. For a comprehensive description see Lu et al. (2016).

### 2.5. The doubly stochastic projected fixed-point (DSPFP) algorithm

The DSPFP algorithm provides an approximate solution to the GM and sub-GM problems. In its original formulation (see Lu et al., 2016), the algorithm accounts for the similarity between both the weights of corresponding edges and the attributes of corresponding vertices. For sake of simplicity, here we omit the part pertaining vertex attributes, since it does not play a role in the proposed application of tractogram alignment^5^. In the following we report the main steps to describe DSPFP.

The minimization problem of Equation (3) can be recast as the maximization problem

(4) $$\begin{array}{l} P^{*}=\underset{X}{\text{argmax}}\frac{1}{2}tr\left(X^{⊤}AX^{⊤}B\right) \end{array}$$

$$\begin{array}{l} \text{s}.\text{t}.\;X1=1,X^{⊤}1=1,x_{ij}\in \left\{0,1\right\} \end{array}$$

see for example Lu et al. (2016), Appendix A, for the complete derivation of Equation (4). By relaxing the constraint that *x*_*ij*_ ∈ {0, 1} into *x*_*ij*_ ≥ 0, the original discrete space of the partial permutation matrices becomes the continuous one of doubly stochastic matrices. The quadratic maximization problem on this continuous space can be addressed by means of the projected fixed-point method, which is iterative:

(5) $$\begin{array}{l} X^{t+1}=\left(1-\alpha \right)X^{t}+\alpha \Pi \left(\nabla f\left(X^{t}\right)\right) \end{array}$$

where α is the step size, Π(·) is the doubly stochastic projection, *f*(*X*) is the target function to be maximized, i.e., $f\left(X\right)=\frac{1}{2}tr\left(X^{⊤}AX^{⊤}B\right)$, so ∇*f*(*X*) = *AXB*. The projection method adopted in DSPFP is based on a recent closed-form solution of doubly stochastic projection, proposed in Zass and Shashua (2006), which is solved by iterating two successive projections until convergence: Π(*X*) = …Π_2_Π_1_Π_2_Π_1_(*X*), where^6^:

(6) $$\begin{array}{l} \Pi_{1}\left(X\right)=X+\left(\frac{1}{N_{A}}I+\frac{1^{⊤}X1}{N_{A}^{2}}-\frac{1}{N_{A}}X\right)11^{⊤}-\frac{1}{N_{A}}11^{⊤}X \end{array}$$

and

(7) $$\begin{array}{l} \Pi_{2}\left(X\right)=\frac{X+|X|}{2}. \end{array}$$

The resulting procedure is reported in Algorithm 1, where *Y* is a *N*_*A*_ × *N*_*A*_ matrix, assuming *N*_*A*_ ≥ *N*_*B*_. The initialization of *X* and *Y* recommended in Lu et al. (2016) is such that *x*_*ij*_ = 1/(*N*_*A*_*N*_*B*_) and *y*_*ij*_ = 0, ∀*i, j*.

**Algorithm 1:**
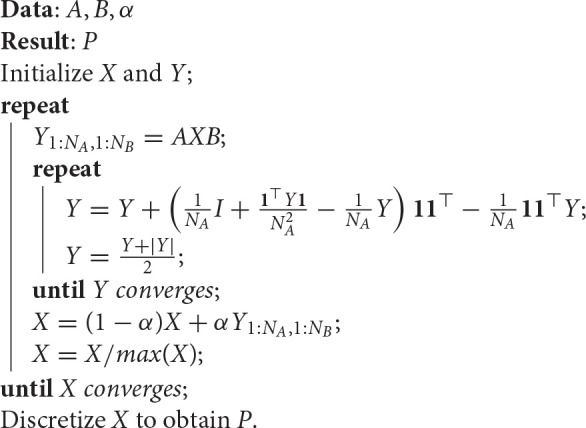
**Doubly Stochastic Projected Fixed-Point (DSPFP), from Lu et al. (2016)**.

The *discretization* of X, required in the last step of Algorithm 1, is a linear assignment problem, which can be implemented with the Hungarian method or approximated with a faster greedy algorithm for the linear assignment method, such as the one used in Leordeanu and Hebert (2005). This greedy approach comprises the steps described in Algorithm 2.

**Algorithm 2:**
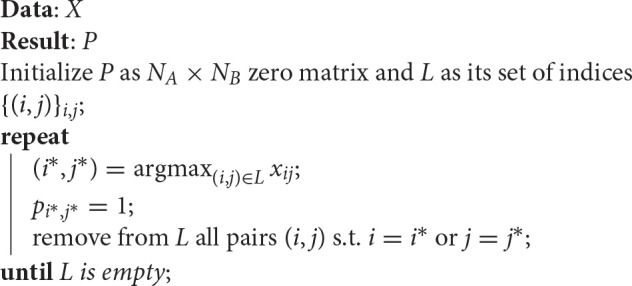
**Greedy algorithm for the linear assignment problem**.

## 3. Experiments

In this section, we describe the experiments to support the claims about the advantages of tractogram correspondence over registration methods for tractogram alignment. The experiment was conducted on the Human Connectome Project (HCP) dMRI dataset, see Van Essen et al. (2013), Sotiropoulos et al. (2013), and Glasser et al. (2013). From that, we extracted data of 10 random subjects, single shell (b = 1000), with the original voxel size (isotropic, 1.25 mm) and 90 gradients. Reconstruction was computed using the constrained spherical deconvolution algorithm (CSD, see Tournier et al., 2007) and tracking was based on the Euler Delta Crossing method (EuDX, see Garyfallidis et al., 2014), with 10^6^ seeds. The resulting tractograms consist of approximately 100–140 thousands streamlines, as reported in Table 1, third and fourth columns.

**Table 1:** **All results of tractograms alignment with FLIRT, ENT, SLR, FNIRT, and the proposed GM across all pairs of subjects**.

**ID subj.A**	**ID subj.B**	**|*T*_*A*_|**	**|*T*_*B*_|**	***J*_*FLIRT*_**	***J*_*ENT*_**	***J*_*SLR*_**	***J*_*FNIRT*_**	***J*_*GM*_**
100307	124422	117354	107891	0.26	0.27	0.26	0.43	**0.59**
	161731	117354	105490	0.33	0.32	0.36	0.44	**0.64**
	199655	117354	134759	0.21	0.24	0.25	0.44	**0.62**
	201111	117354	145364	0.18	0.19	0.18	0.45	**0.51**
	239944	117354	116598	0.22	0.23	0.23	0.47	**0.63**
	245333	117354	106215	0.22	0.20	0.24	0.45	**0.62**
	366446	117354	138380	0.19	0.21	0.20	0.47	**0.61**
	528446	117354	123856	0.16	0.10	0.25	0.44	**0.53**
	856766	117354	116532	0.25	0.26	0.27	0.45	**0.59**
124422	161731	107891	105490	0.28	0.27	0.28	0.42	**0.56**
	199655	107891	134759	0.19	0.20	0.21	0.41	**0.46**
	201111	107891	145364	0.21	0.20	0.21	0.44	**0.54**
	239944	107891	116598	0.26	0.19	0.26	0.45	**0.56**
	245333	107891	106215	0.26	0.28	0.29	0.44	**0.54**
	366446	107891	138380	0.19	0.17	0.24	0.44	**0.59**
	528446	107891	123856	0.19	0.17	0.27	0.44	**0.55**
	856766	107891	116532	0.24	0.23	0.25	0.42	**0.60**
161731	199655	105490	134759	0.17	0.21	0.24	0.41	**0.53**
	201111	105490	145364	0.14	0.17	0.19	0.43	**0.48**
	239944	105490	116598	0.23	0.24	0.24	0.42	**0.58**
	245333	105490	106215	0.28	0.27	0.29	0.45	**0.57**
	366446	105490	138380	0.16	0.11	0.20	**0.45**	0.43
	528446	105490	123856	0.18	0.24	0.27	0.44	**0.54**
	856766	105490	116532	0.23	0.22	0.27	0.44	**0.58**
199655	201111	134759	145364	0.25	0.25	0.23	0.43	**0.57**
	239944	134759	116598	0.23	0.17	0.30	0.42	**0.65**
	245333	134759	106215	0.18	0.10	0.26	0.44	**0.58**
	366446	134759	138380	0.30	0.28	0.30	0.43	**0.62**
	528446	134759	123856	0.26	0.30	0.30	0.43	**0.58**
	856766	134759	116532	0.25	0.22	0.27	0.40	**0.63**
201111	239944	145364	116598	0.25	0.29	0.27	0.41	**0.58**
	245333	145364	106215	0.20	0.25	0.27	0.44	**0.57**
	366446	145364	138380	0.33	0.35	0.33	0.44	**0.64**
	528446	145364	123856	0.29	0.10	0.31	0.43	**0.58**
	856766	145364	116532	0.27	0.28	0.28	0.42	**0.54**
239944	245333	116598	106215	0.24	0.23	0.24	0.42	**0.53**
	366446	116598	138380	0.21	0.22	0.25	0.40	**0.57**
	528446	116598	123856	0.17	0.22	0.24	0.39	**0.47**
	856766	116598	116532	0.20	0.17	0.24	0.41	**0.58**
245333	366446	106215	138380	0.14	0.23	0.22	0.41	**0.54**
	528446	106215	123856	0.17	0.25	0.24	0.44	**0.60**
	856766	106215	116532	0.20	0.25	0.26	0.41	**0.55**
366446	528446	138380	123856	0.29	0.28	0.32	0.45	**0.54**
	856766	138380	116532	0.27	0.23	0.28	0.44	**0.62**
528446	856766	123856	116532	0.22	0.19	0.27	0.45	**0.61**
average over all 45 pairs				0.23	0.22	0.26	0.43	**0.57**
standard dev. over all 45 pairs				±0.05	±0.06	±0.03	±0.02	±0.05

*In each row are indicated the pairs of subjects IDs, the size of the two tractograms and the five degrees of overlap after alignment,in terms of Jaccard index (J), averaged over nine tracts. The averages and standard deviations over all 45 pairs are reported in the last row*.

*This table reports the results of the comparison of five different methods across multiple pairs of subjects. In each row, the score of each of the five methods is reported: J_FLIRT_, J_ENT_, J_SLR_, J_FNIRT_, J_GM_. In each row, the higher the value, the better. The number in bold face is the highest value among the five values in the same row, i.e., it indicates the best method for each pair of subjects [highest overlap score (J) for each pair of subjects]*.

In order to quantify the quality of tractogram alignment produced by the methods in the literature and by the proposed one, we relied on measuring the degree of overlap of corresponding anatomical bundles/tracts after whole tractogram alignment (see Golding et al., 2011; Garyfallidis et al., 2015). Since the bundle/tract information is never used to compute alignments, we can safely assume that, the better such overlap, the better the alignment of tractograms. In the following, we provide details of the set of alignment methods used in the comparison and of the procedure to obtain the bundles/tracts. Then we report the results of all experiments.

### 3.1. Alignment of tractograms

We aligned all pairs of tractograms obtained from the 10 subjects, i.e., 45 pairs. We compared the following alignment methods: standard affine registration of voxels, affine registration of streamlines, non-linear registration of voxels and the proposed method based on graph matching (GM). Voxel-level affine registration was computed with FSL/FLIRT on the B0 images. Streamline affine registration was computed with two different algorithms: the entropy-based group-wise registration (ENT) algorithm^7^ from O'Donnell et al. (2012) and with the streamline linear registration (SLR) algorithm^8^ from Garyfallidis et al. (2015). In all cases, affine registration was applied to streamline coordinates using the function dipy.tracking.streamline.apply_affine(), from DiPy. Non-linear voxel-level registration was computed with FSL/FNIRT, using the deformation fields directly provided within the HCP dataset^9^. GM-based alignment was computed using the DSPFP algorithm described in Section 2.5 and implemented by us in Python language. Our implementation is available under a Free/OpenSource license at: https://github.com/emanuele/DSPFP.

In order to quantitatively compare the five different whole-tractogram alignment methods we followed the common practice, also described in Garyfallidis et al. (2015) and Golding et al. (2011), i.e., we computed the overlap in voxels of a set of corresponding anatomical tracts after the whole brain alignment is done. Before alignment, we segmented a set of tracts in each tractogram with the white matter query language (WMQL, see Wassermann et al., 2013)^10^ and then used the streamline IDs to obtain the tracts on the tractogram after alignment. The set of tracts comprised: Cingulum Bundle (left and right), Inferior Occipito Frontal Fascicle (left and right), Uncinate Fascicle (left and right), Arcuate Fascicle (left), and Corpus Callosum (part 2 and 7). We selected these specific nine tracts because their segmentation, obtained through WMQL, was more consistent across subjects, in terms of number of streamlines, see Supplementary Material for additional details. In Supplementary Material, we extend also this analysis to include further bundles/tracts, which are known to have high variability across subjects. The fraction of overlapping voxels between the tract ($t_{A}^{al}$) of the aligned tractogram and the one (*t*_*B*_) in the target tractogram, usually called Jaccard index *J*, is then:

(8) $$\begin{array}{l} J=\frac{\left|v\left(t_{A}^{al}\right)\cap v\left(t_{B}\right)\right|}{\left|v\left(t_{B}\right)\right|} \end{array}$$

where *v*(*t*) indicates the set of voxels crossed by the streamlines of tract *t*, while |·| is the set size.

### 3.2. Graph matching: further approximation

**Figure 2:**
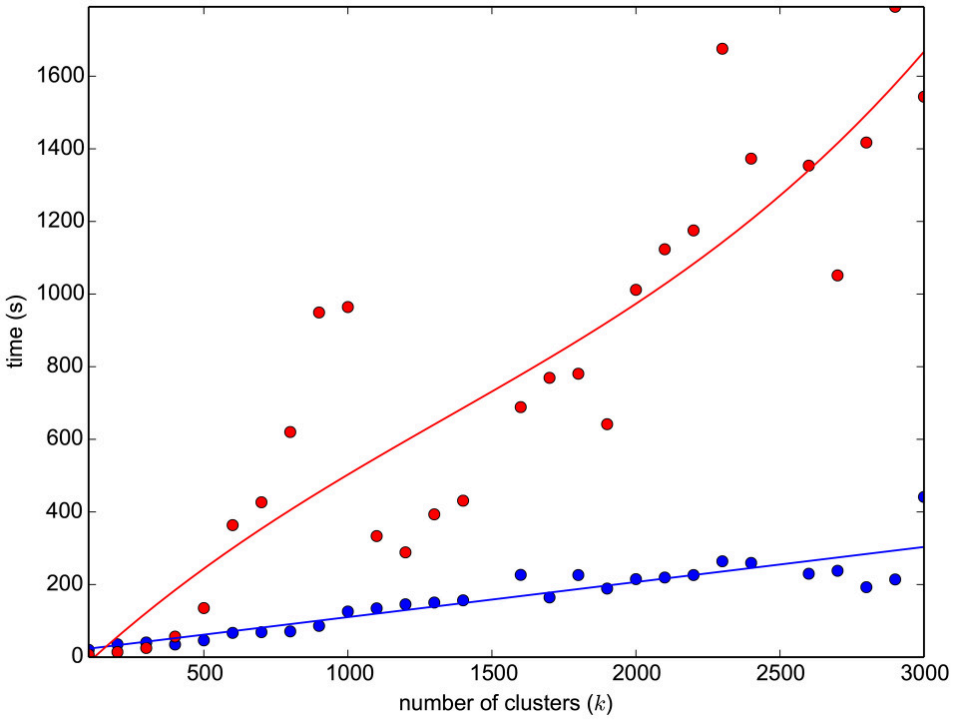
**The graph reports the time required to simplify a 110K-streamlines tractogram (HCP subject ID:100307) with the mini-batch ***k***-means (blue dots, plus linear interpolation), as a function of the number of clusters**. It reports also the time required to run graph matching (DSPFP, red dots, plus cubic interpolation) between two simplified graphs, each of *k* streamlines/vertices, of two subjects (HCP ID1:100307, ID2:124422).

As mentioned in Section 2.4, one limitation of the algorithms for approximate graph matching is the high computational cost when the number of nodes exceeds a few thousands. Specifically, for the adopted DSPFP, see Figure 2 for the exact timing to perform graph matching on sets of streamlines till size 3000. The computational and storage resources necessary to handle the matching of graphs generated from an entire tractogram, i.e., *n* = 10^5^ nodes and 10^10^ edges, are beyond the capability of modern computers. For this reason, in order to obtain the correspondence between tractograms, we introduced a further approximation with a simple three-steps procedure, similar to the one proposed for the SLR algorithm in Garyfallidis et al. (2015):

1. We clustered each tractogram into a given number of clusters (*k* = 1000) and defined the median (centroid) streamline as the *representative* for each cluster. We adopted the fast mini-batch *k*-means algorithm described in Olivetti et al. (2013) and Porro-Muñoz et al. (2015).
2. We computed graph matching between the two graphs made only with the *k* representatives, in order to obtain corresponding clusters.
3. For each pair of corresponding clusters, we computed the graph matching between their streamlines, in order to find streamline correspondence.

Notice that, in all cases, approximate graph matching was computed with the DSPFP algorithm (see Section 2.5), with random initialization, i.e., *x*_*ij*_ ~ *U*[0, 1]. This is different from the initialization recommended in Lu et al. (2016), i.e., *x*_*ij*_ = 1/(*N*_*A*_*N*_*B*_), but we observed failure of convergence in a few cases with such constant value, while random initialization always converged. We also tried the initialization resulting from an initial affine registration (based on SLR) and we obtained very similar results. For generality of the proposed method, we prefer to recommend random initialization.

The steps introduced above aim at simplifying the original tractograms, in order to reduce the computational problem of graph matching. Other choices can be made, for example using different clustering algorithms, see for example Garyfallidis et al. (2012), or recent specific methods for tractogram simplification, like Gori et al. (2016). The motivation of our choice, for adopting the algorithm described in Olivetti et al. (2013), is eminently computational. The time required to carry out the simplification, which is in the order of a couple of minutes, see Figure 2, is substantially inferior to that of other algorithms in the literature.

### 3.3. Results

Performing the three-steps procedure required approximately 30 min of computation for each pair of tractograms on a standard desktop computer. In Figure 2, we report the time to compute mini-batch *k*-means for streamlines on a full tractogram^11^ Linear registration required a few minutes of computation for FLIRT and SLR and approximately 30 min for ENT. FNIRT deformation fields were already available within the HCP dataset, anyway they usually require a few minutes of computation.

Table 1 shows the results of all 45 pairs across the five different alignment methods, averaged over nine tracts. The first two columns report the HCP subject identifiers of the two tractograms considered in the alignment. The third and fourth columns report the size of the tractograms of the two subjects in terms of number of streamlines. The remaining five columns report the degree of overlap between the aligned tractograms (*J*, higher is better), averaged over the NINE tracts considered, for the five methods considered in the comparison: FLIRT, ENT, SLR, FNIRT, and GM. For each pair of subjects, the highest degree of overlap is indicated in bold face. In order to avoid unreasonable outliers, we filtered out the cases for which the difference in number of streamlines for the same tract was extreme, i.e., > 60%, denoting problems in the WMQL segmentation. See Supplementary Material for an analysis of this issue.

**Figure 3:**
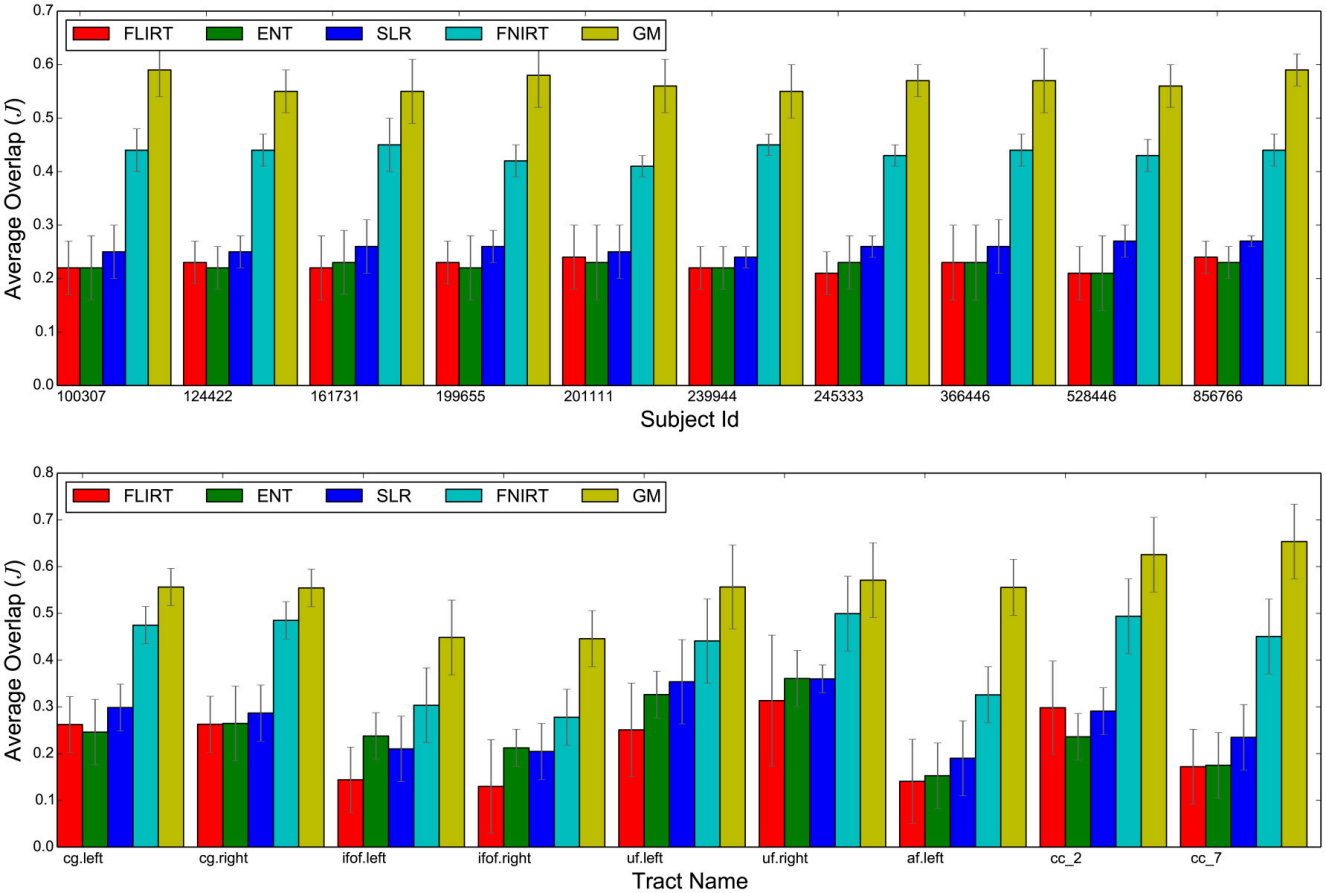
**Summary of the results: average overlap between corresponding tracts after whole brain alignment for the FLIRT, ENT, SLR, FNIRT, and the proposed GM**. The upper bar-graph shows the overlap for each subject averaged over all nine tracts and the remaining nine subjects. The lower bar graph shows the overlap for each tract averaged over all pairs of subjects.

Figure 3 reports a visual aggregated summary of the results of the experiments. In the upper graph, for each subject, five barplots are reported. Each barplot indicates the overlap of tractograms after alignment (*J*, higher is better) averaged over the nine pairs that include that subject and all nine tracts. The graph in the lower part of Figure 3 shows the results for each tract, where the overlap for each of the five methods in the comparison is averaged over the 45 pairs of subjects.

**Figure 4:**
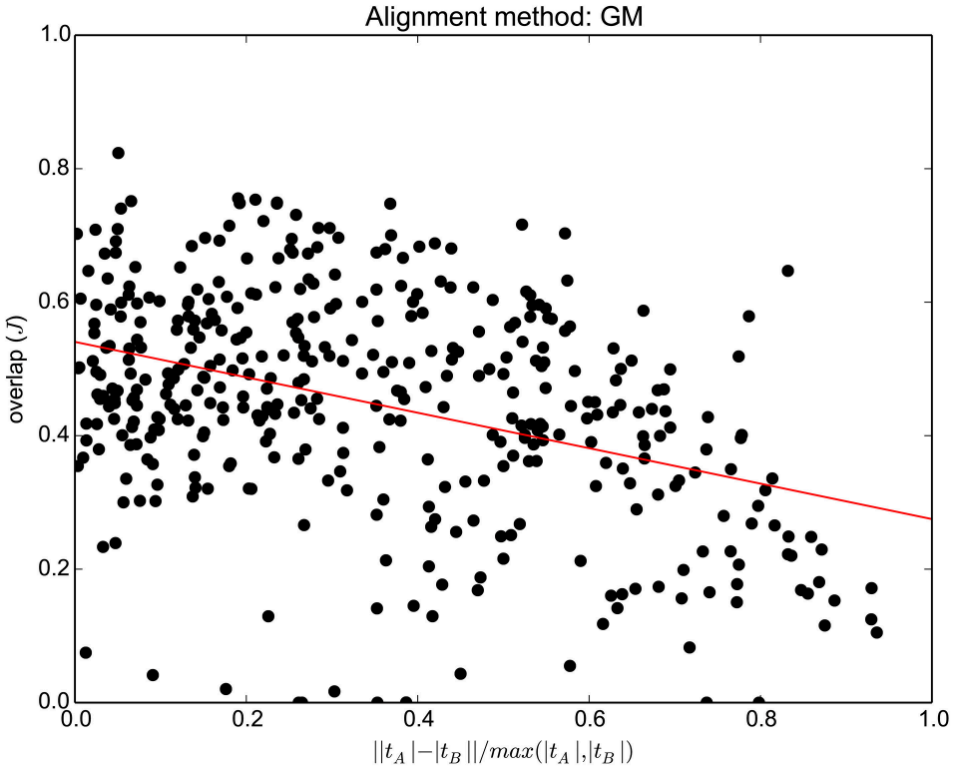
**For each of the 45 pairs of subjects and 9 tracts/bundles (45 × 9 = 405 points in total), the graph shows the tract overlap after whole tractogram alignment performed with GM, as a function of the difference between that tract across the two subjects**. The difference, in number of streamlines, is quantified as $\Delta_{AB}=\frac{\left||t_{A}|-|t_{B}|\right|}{max\left(\left|t_{A}|,|t_{B}\right|\right)}$.

In order to show the performance of the proposed method, with respect to the difference between the same bundle in two different subjects, in Figure 4 we report the quality of alignment (*J*_*GM*_) as a function of the difference between |*t*_*A*_| and |*t*_*B*_|, where |*t*| is the number of streamlines of tract/bundle *t*. Such difference is quantified as: $\Delta_{AB}=\frac{\left||t_{A}|-|t_{B}|\right|}{max\left(\left|t_{A}|,|t_{B}\right|\right)}$, which is 0 for bundles with the same number of streamlines and increases up to one according to how much the two sizes differ. Each point represents one tract for one pair of subjects, e.g., Cingulum left for the pair HCP ID:100307 and HCP ID:124422. Thus, there are 45 × 9 = 405 points in total. In Supplementary Material we report the equivalent graphs for the other alignment methods in the comparison.

**Figure 5:**
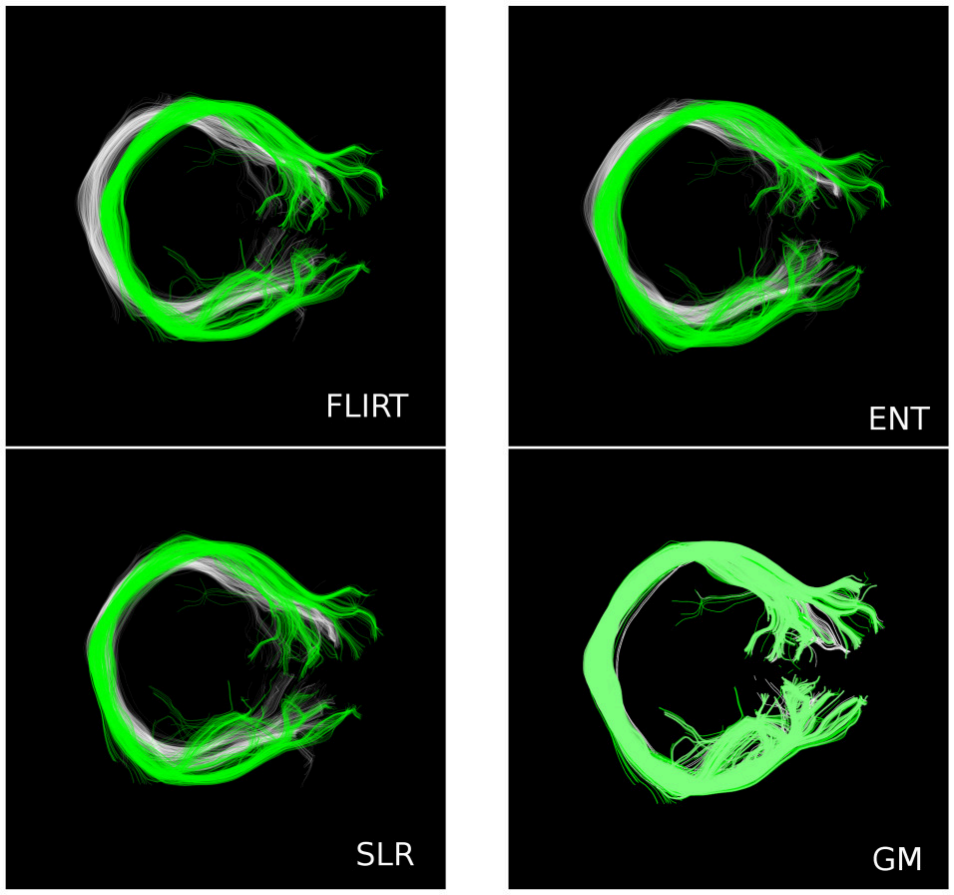
**A paradigmatic example where there is systematic displacement between the corresponding tract (CC, Section 7) of two subjects after whole-brain affine registration, with FSL/FLIRT, ENT and SLR**. Conversely, GM is able to provide much better alignment.

In Figure 5 we show one paradigmatic example where there is a systematic displacement between the corresponding tracts (Corpus Callosum, Section 7, as defined by the WMQL) of two subjects (HCP ID1: 100307, ID2: 199655) after whole-brain affine registration. In white and green we show the two tracts after the registration computed with FSL/FLIRT, ENT, and SLR^12^. In the same figure, we also report the alignment computed with GM, which shows a much superior match because GM acts also at the local level. For this specific example, the fractions of overlapping voxels for that tract are: *J*_*FLIRT*_ = 0.18, *J*_*ENT*_ = 0.26, *J*_*SLR*_ = 0.24, *J*_*FNIRT*_ = 0.52, and *J*_*GM*_ = 0.81.

### 3.4. A technical comment about ENT

As reported in the last row of Table 1, we noticed that the ENT algorithm, from O'Donnell et al. (2012), exhibited substantially higher variance in the results with respect to other algorithms and in particular with respect to the SLR algorithm, which is the most similar one. We explored this issue and, from preliminary experiments, we believe that such increased variance is mainly due to the way in which the tractograms are simplified, in order to reduce the amount of computation. In ENT, a random subsample of streamlines for each tractogram is used, while in SLR the subsample is the set of representatives of the clusters. The random subsample of ENT is of 20000 streamlines^13^ and then it is repeatedly reduced within that algorithm. Conversely, SLR registration is based on 2000–3000 representatives, see Garyfallidis et al. (2015). Additionally, ENT is optimized for the joint alignment of multiple subjects. All these facts contributes to the explanation of the observed increased variance^14^.

## 4. Discussion and conclusions

In this work, we present whole brain tractogram alignment based on computing streamline correspondence across subjects as a graph matching problem. This work is the first one that presents a quantitative comparison across streamline-based alignment methods for tractograms. Table 1 and Figure 3 clearly show that tractogram correspondence, implemented as graph matching (GM), is able to align tractograms much better than what global affine registration can do, both voxel-based and streamline-based. The overlap, in terms of voxels, of the aligned tracts with respect to the target tracts is two times better with the proposed method than with affine methods. This occurs uniformly across all subjects and all tracts. We also notice that, as expected, all the affine-based registration methods exhibit comparable results between each other, within the reported standard deviations, meaning that there is an inherent limitation in affine transformations, irrespective of the loss function they optimize.

Such a positive result for GM against affine methods is not surprising, because it is expected that one global linear transformation cannot reconcile local systematic differences between white matter bundles across subjects. Differently, GM is designed to optimize the match both at the global and local level. This aspect is clearly exemplified in Figure 5, where some notable displacement between the tracts remains after global affine alignment, but not in the case of GM. The locality of GM and, more in general, of the correspondence-based alignment, act as if each streamline in one tractogram is deformed to exactly match the corresponding streamline in the other tractogram. This corresponds to a different deformation for each streamline. The global aspect is also preserved because GM is a joint optimization problem across all vertices/streamlines.

The results in Table 1 show also the quality of alignment obtained through non-linear registration of volumes, by means of FSL/FNIRT (eighth column). Since non-linear registration is able to operate local changes, the quality of such alignment is much better than that of linear methods, as expected. By comparing the results of GM and FNIRT, we observe that the quality of GM is still superior, i.e., *J*_*FNIRT*_ = 0.43 ± 0.02 vs. *J*_*GM*_ = 0.57 ± 0.05, even though not by a large margin. Our interpretation of this result is that FNIRT optimized the match of the T1 images, which contain white matter boundaries but not the detailed structure, while our GM-based alignment procedure optimized the match of streamlines. Since the evaluation is based on corresponding anatomical white matter tracts, our proposed method has an advantage, that may explain part of difference in the results. It is true that non-linear volumes-based alignment can target other kinds of volumes that incorporates dMRI information, like FA (fractional anisotropy) or B0. For this reason, further experiments are needed in order explore this comparison. Nevertheless, a main difference remain: the proposed method operates on streamlines, which is the focus of this paper, while the non-linear volume-based algorithms, like FNIRT, operate on voxel-level information.

The quality of whole brain alignment provided by GM is expected to decrease when the difference in number of streamlines between corresponding bundles is large. In Figure 4, the trend line shows this expected decay, at the level of individual pairs of bundles. Nevertheless we can also expect that, given one bundle, large differences do not occur between all pairs of subjects. This is confirmed in Figure 3 (lower part), where standard deviations are moderate. This is further confirmed in Supplementary Materials, where the result is extended to SLF II (right) and MDLF (right and left), which are known to be highly variable across subjects. Nevertheless, we have also to take into account the limitations of the ground truth used in this study, which is based on automatic bundle segmentation, see Wassermann et al. (2013). The limitations of such segmentation are explored in Supplementary Materials and may contribute to the variability of the results observed in Figure 4. The use of expert-based segmentation might partly mitigate this problem.

The proposed GM algorithm, despite multiple approximations, can recover a substantial portion of what other methods miss. It has to be noted that this improvement comes at the cost of a substantial increase in the time of computation, at least with respect to FLIRT, SLR, and FNIRT. The time required by the proposed implementation of the GM-based algorithm is ten times more than that required by these algorithms. The total amount of time required by the proposed method is a function of the number of clusters (*k*) chosen during the clustering-based approximation, as illustrated in Figure 2. Specifically, it is composed by the time to obtain the clustering, the time to compute the graph matching between cluster representatives and the time for graph matching between the streamlines of corresponding clusters. Notice that the proposed clustering-based approximation could be used for addressing the scalability issues of other algorithms, such as the one of Durrleman et al. (2011). Future work has to be done in this direction.

Despite clearly positive results, the solution proposed in this work suffers various limitations, that provide ground for interesting future work. At a general level, the idea of putting in correspondence streamlines across tractograms may be limited by the quality of the tractography algorithms, topic on which there is still major debate. Moreover, finding the correspondence for *all* streamlines across tractograms may be challenged because, for example, artifactual streamlines should not have a corresponding one. A filtering mechanism to avoid such cases is currently not available. In the same way, at the implementation level, the sub-optimal constraint of one-to-one correspondence of GM may be excessive in some cases. Future work should address the relaxation of such constraint. At the application level, aligning tractograms for transferring anatomical knowledge to a new subject, e.g., for segmentation of bundles, is the straightforward application of the proposed method. Despite the usefulness of such task, it is still not clear how to address other common tasks, like alignment at the group-level and atlas construction, on which we are focusing our future work. From this point of view, the idea of exploiting the correspondence between streamlines opens up new directions of research and opportunities for improvement in neuroscientific and clinical applications.

## Author contributions

EO conceived the ideas, designed the experiments, conducted the experiments, and wrote the manuscript. NS contributed to the design of the experiments, conducted the experiments, and contributed to the manuscript. PA contributed to the ideas and to the manuscript.

### Conflict of interest statement

The authors declare that the research was conducted in the absence of any commercial or financial relationships that could be construed as a potential conflict of interest.

## Footnotes

^1^In the whole paper we use *alignment* as the generic term to indicate the task of putting in correspondence two anatomical structures. We use the term *registration* to specifically indicate the transformation-based methods to obtain alignment.

^2^Technically, each vertex is an object whose property is the identifier of the streamline to which it is associated.

^3^Historically, the GM problem refers to finding the vertices of *G*_*B*_ that match those of *G*_*A*_, and not the contrary, which is the natural way to formulate the alignment problem.

^4^All these algorithms are iterative, with some sort of convergence criteria.

^5^Technically, this means simply to set to zero the matrix *K* in all equations in Lu et al. (2016).

^6^**1** indicates the vector with all elements equal to one.

^7^We adopted the software implementation provided by the authors of ENT, available at: https://github.com/ljod/whitematteranalysis.

^8^As implemented in DiPy, http://nipy.org/dipy.

^9^The estimation of the deformation fields of the HCP dataset is described in Glasser et al. (2013) in the following way: “After bias field correction of the T1w and T2w images, the T1w image is registered to MNI space with a FLIRT 12 DOF affine and then a FNIRT non-linear registration, producing the final non-linear volume transformation from the subject's native volume space to MNI space.”

^10^According to the white matter parcellation provided by the HCP initiative.

^11^reproducing the results in Olivetti et al. (2013). and the time for DSPFP, for various *k*.

^12^FSL/FNIRT is missing from this visual example because we did not apply the resulting deformation field to the streamlines: such procedure is not sufficiently analyzed in the literature. Nevertheless, we report the quantitative results at voxel-level.

^13^According to the guidelines in https://github.com/ljod/whitematteranalysis.

^14^We thank Reviewer 2 for insightful comments on this part.
