# Supplementary material for: Alignment of Tractograms As Graph Matching
Source: Front Neurosci. 2016 Dec 5;10:554. doi: 10.3389/fnins.2016.00554 (PMC5136564; doi:10.3389/fnins.2016.00554)
Supplement: Supplementary file 1 [file DataSheet1.pdf]

Supplementary Material: Alignment of Tractograms as Graph Matching

Emanuele Olivetti, Nusrat Sharmin and Paolo Avesani
*Correspondence:
Author Name: Emanuele Olivetti
olivetti@fbk.eu

1 TRACTS/BUNDLES WITH HIGH VARIABILITY ACROSS SUBJECTS

We investigated a larger set of tracts/bundles than that described in the paper. In Table S1 we report the size, in number of streamlines, of 18 tracts for each of the 10 subjects considered in our study. Each tract was automatically segmented with the white matter query language (WMQL), see Wassermann et al. (2013). As it is clearly shown, the variability in number of streamlines across subjects changes dramatically from one tract to another. For example the Cingulum left (cg.left, 1st row), has a moderate variability across subjects, around 20%. Differently the Arcuate Fasciculus right (af.right, 10th row) shows extreme variability, e.g. 508 streamlines in subject 5 and ≤ 2 streamlines in subjects 4, 6 and 10. For this reason, we divided the tracts in two groups, of low and high variability. In the upper part of Table S1 we report the tracts with low variability, that were included in the study. In the lower part of Table S1, below the mid line, we report the tracts with very high variability, that were excluded from our study.

It is expected that part of the observed variability is due to anatomical differences across the population. Nevertheless, we believe that part of the reason for the extremes we observe in the lower part of Table S1 is due to poor segmentation and, possibly, also due to limitations in tractography data and white matter parcellation. An expert-made segmentation instead of the automatic one, might reduce the issue. Given this problem, in this work we want to decouple the variability intrinsic to the methods from the variability in the ground truth, which here can be considered as unwanted noise. Such considerations led us not to consider the tracts in the lower part of Table S1 from our study.

In order to enrich the results presented in the paper, here in Supplementary Material we extend the experiments with three more tracts that were previously excluded: SLF II, MDLF left and MDLF right. We chose these tracts because they exhibit less extreme variability among the excluded ones. For these tracts, in Figure S2, we show the average overlap after whole tractogram alignment with FLIRT, ENT, SLR, FNIRT and the proposed GM. The height of each bar in the graph represents the average overlap, computed over the 45 pairs of subjects considered in the study. From those results, we observe that, in general, the overlap of linear methods was lower than the cases reported in the paper, i.e. $J \in [0.10, 0.15]$. FNIRT and GM showed much higher degree of overlap than linear methods and comparable results between each other, i.e. $J \in [0.45, 0.55]$. Nevertheless these results are among the lowest values obtained for other tracts in the paper.

With the three new bundles considered here, in Figure S3, for the proposed GM, we extend Figure 4 of the paper by showing the degree of overlap (J_{GM}) vs. the difference between the corresponding bundles, at the level of individual pairs of subjects (45 pairs) and bundle (now 12 bundles): $45 \times 12 = 540$ points. In Figure S4, we also report the equivalent graphs for FLIRT, ENT, SLR and FNIRT. As expected, in all cases the trend, as linear interpolation (red line), shows the decay of overlap when the corresponding bundle

bundle	subj1 100307	subj2 124422	subj3 161731	subj4 199655	subj5 201111	subj6 239944	subj7 245333	subj8 366446	subj9 528446	subj10 856766
cg.left	1181	905	1235	1326	1361	1185	1230	1126	1396	1150
cg.right	969	1002	974	1036	1247	1178	1050	904	1235	861
ifof.left	169	183	66	142	92	202	54	290	161	68
ifof.right	152	69	20	284	131	176	40	311	22	109
uf.left	138	193	179	166	281	60	144	296	102	66
uf.right	250	158	195	244	211	73	145	182	156	41
cc_7	546	431	337	442	413	247	441	577	520	319
cc_2	661	283	300	302	670	407	314	638	464	628
af.left	92	333	273	176	550	124	352	200	234	172
af.right	35	190	172	1	508	2	137	8	77	0
slf1.left	11	0	0	0	0	0	0	0	0	0
slf1.right	22	1	8	27	0	20	0	16	18	3
slf2.left	193	64	0	130	30	36	0	0	0	1
slf2.right	257	22	25	182	67	1	62	79	74	20
slf3.left	4	0	0	0	0	0	0	0	0	0
slf3.right	0	0	1	2	19	0	43	71	18	0
mdlf.left	55	116	137	69	16	11	299	239	227	22
mdlf.right	256	40	146	27	113	244	13	128	16	27

Figure S1. Tract/Bundle sizes, in terms of number of streamlines across the 10 subjects considered in this study. The Tracts were segmented with WMQL, see Wassermann et al. (2013).

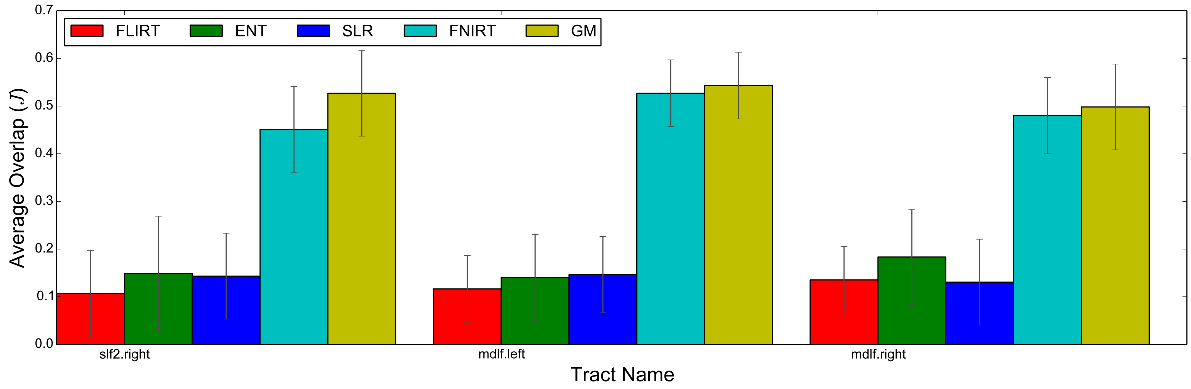

Figure S2. additional tracts

across two subjects greatly differ in number of streamlines. These graphs also show that GM and FNIRT align tractograms much better than FLIRT, ENT and SLR.

REFERENCES

Wassermann, D., Makris, N., Rathi, Y., Shenton, M., Kikinis, R., Kubicki, M., et al. (2013). On describing human white matter anatomy: the white matter query language. *Medical image computing and computer-assisted intervention : MICCAI ... International Conference on Medical Image Computing and Computer-Assisted Intervention* 16, 647–654

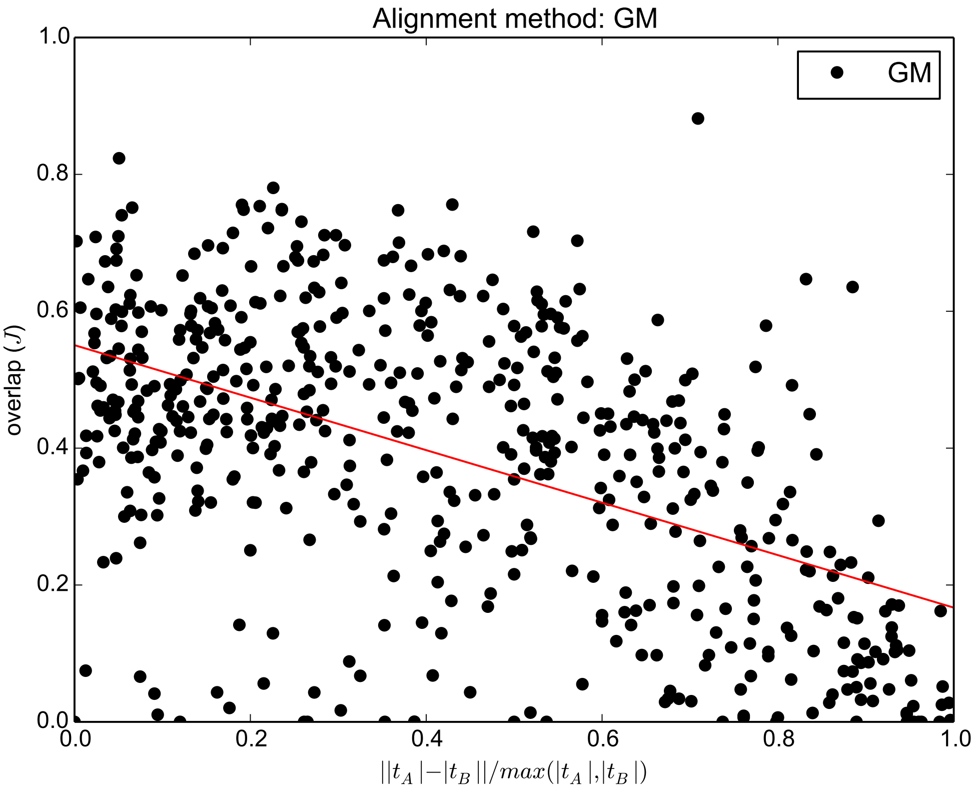

Figure S3. For each of the 45 pairs of subjects and 12 tracts/bundles ($45 \times 12 = 540$ points in total), the graph shows the tract overlap after whole tractogram alignment performed with GM, as a function of the difference between that tract across the two subjects. The difference, in number of streamlines, is quantified as $\Delta_{AB} = \frac{||t_A|-|t_B||}{max(|t_A|,|t_B|)}$.

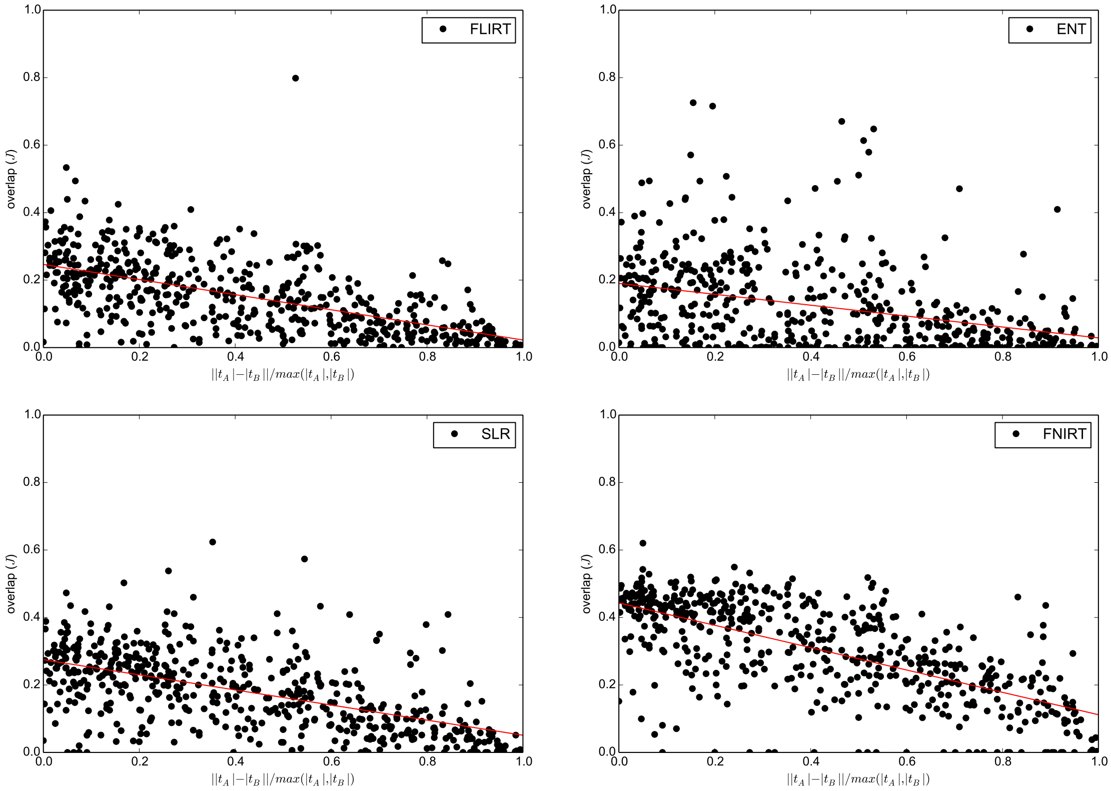

Figure S4. For each of the 45 pairs of subjects and 12 tracts/bundles ($45 \times 12 = 540$ points in total), the graph shows the tract overlap after whole tractogram alignment performed with FLIRT, ENT, SLR and FNIRT, as a function of the difference between that tract across the two subjects. The difference, in number of streamlines, is quantified as $\Delta_{AB} = \frac{||t_A|-|t_B||}{max(|t_A|,|t_B|)}$.
